# Supplementary material for: Landscape of submitochondrial protein distribution
Source: Nat Commun. 2017 Aug 18;8:290. doi: 10.1038/s41467-017-00359-0 (PMC5561175; doi:10.1038/s41467-017-00359-0)
Supplement: Supplementary file 1 — Supplementary Info [file 41467_2017_359_MOESM1_ESM.pdf]

File Name: Supplementary Information
Description: Supplementary Figures, Supplementary Table and Supplementary References

File Name: Peer Review File
Description:

File Name: Supplementary Data 1
Description: Searchable map of mitochondrial protein distribution. Proteins are assigned into integral membrane (green), peripheral membrane (orange) and soluble (blue) protein categories.

File Name: Supplementary Data 2
Description: Raw data of the four data sets of SNcarb/PELcarb and SNson/PELson ratios and determination of Yeast/Mito ratios of identified proteins.

File Name: Supplementary Data 3
Description: List of novel proteins identified in highly purified mitochondria.

File Name: Supplementary Data 4
Description: Identification of integral inner and outer membrane proteins. OMLight to TOTHeavy ratios were determined and calculated as relative intensities for proteins in the OM fraction. Proteins with a rel. intensity > 25 were considered OM and < 25 as inner membrane proteins.

File Name: Supplementary Data 5
Description: Integrated map of submitochondrial protein distribution. Annotation of proteins was based on the integration of the average values of SNcarb/PELcarb and SNson/PELson ratios and the reference proteomes of outer membrane (resident peripheral and integral proteins) and the soluble IMS proteins (see Online Methods). Proteins residing slightly outside of respective cluster regions were assigned but indicated in red colour. Yeast/Mito ratios indicate presence of respective protein in either only mitochondria or additional cellular compartments. In a few cases of obtaining only one of the two SILAC ratios, high SNson/PELson or low SNcarb/PELcarb ratios were assigned as soluble or integral, respectively (indicated in red colour). --, ratio could not be determined; *, no SILAC ratio determined as the protein was absent either in heavy or light sample. Upon manual inspection the protein was set to either 20-fold up or down-regulated (i.e. 20.00 or 0.05); **, same, but only based on one replicate. Λ= likelihood estimators for the soluble (sol), peripheral (per) and integral (int) models.

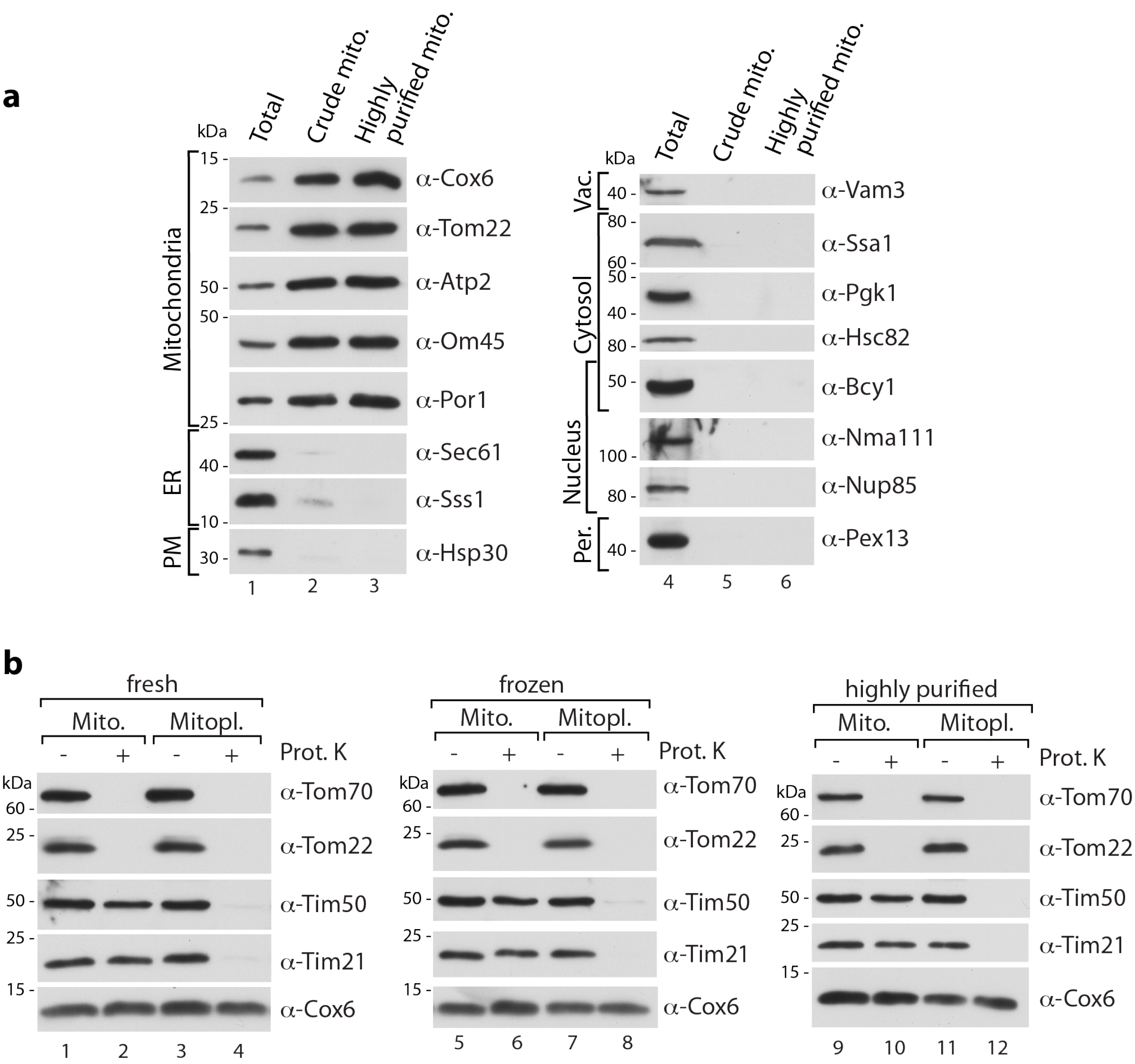

Supplementary Figure 1 | Quality control of mitochondrial integrity. (**a**) Immunoblot analysis of total yeast cells (Total), crude mitochondria and highly purified mitochondria. ER, endoplasmic reticulum; PM, plasma membrane; Vac., vacuole; Per., peroxisome. (**b**) Western blot analysis on the integrity of isolated mitochondria under iso- (Mito.) or hypoosmotic (Mitopl.) conditions upon addition of Proteinase K (Prot. K). Mitochondria were treated directly after isolation (fresh), freeze-thawing (frozen) or after gradient purification followed by freeze thawing (highly purified). Accessibility of the IMS-exposed domains of the inner membrane proteins Tim21 and Tim50 serve as quality control for mitochondrial integrity[1,2].

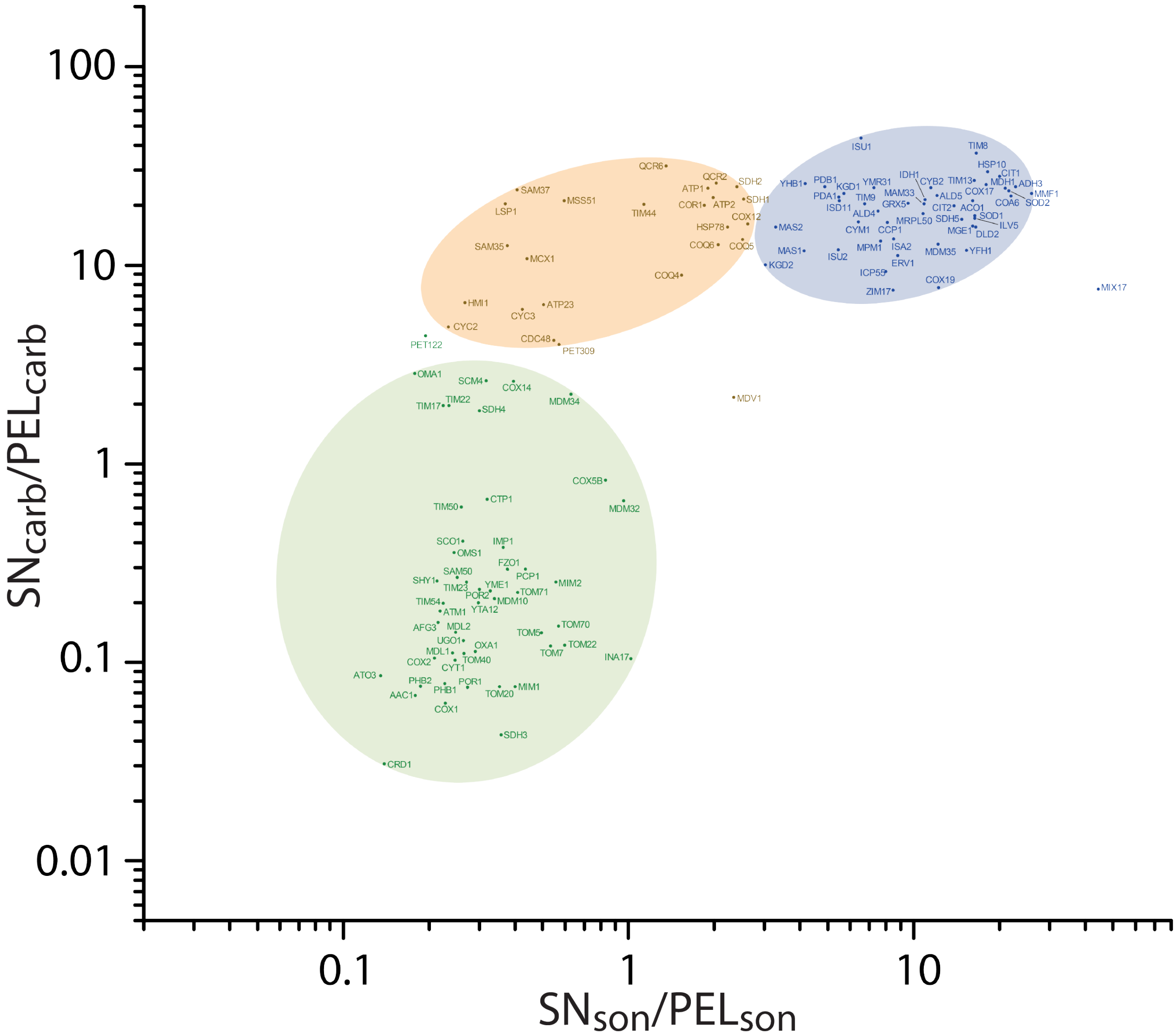

Supplementary Figure 2 | Mapping of mitochondrial protein reference set. Map of SN_{carb}/PEL_{carb} and SN_{son}/PEL_{son} SILAC ratios for a set of reference proteins with clear reported annotation. Proteins from different classes localize to distinct clusters representing integral membrane, peripheral membrane and soluble proteins.

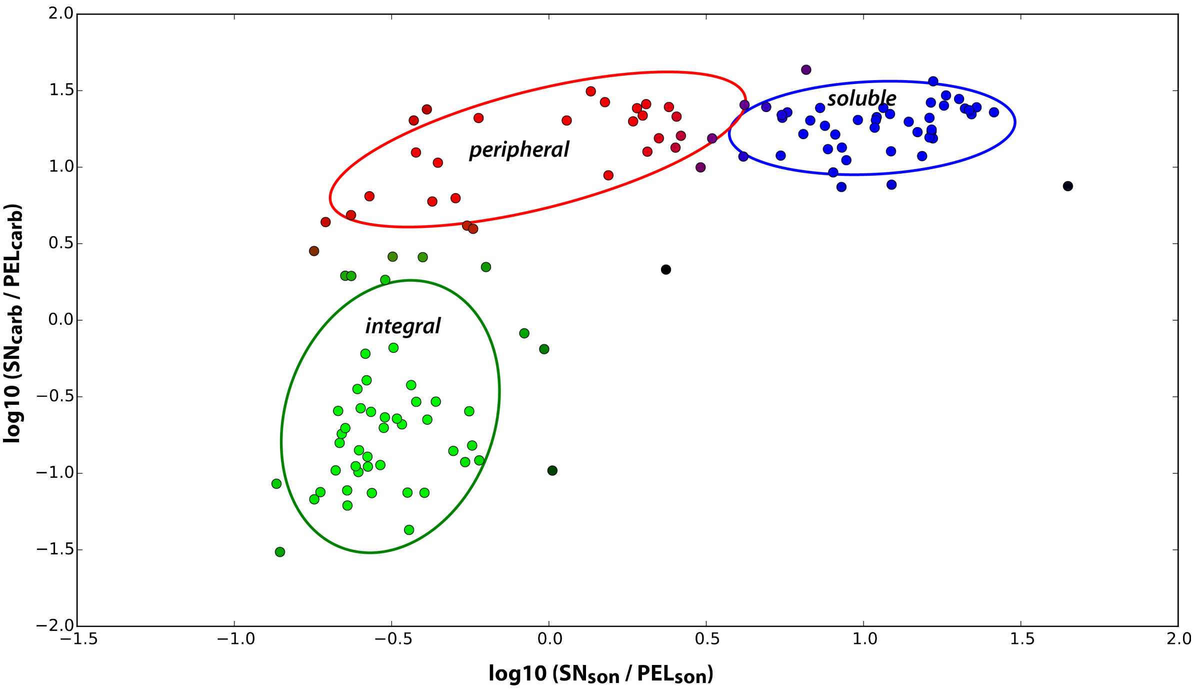

Supplementary Figure 3 | Distribution of the reference protein set P_L. For every protein, all 3 color channels RGB were multiplied by their corresponding probabilities to indicate memberships. The model boundaries indicate 80% of each density.

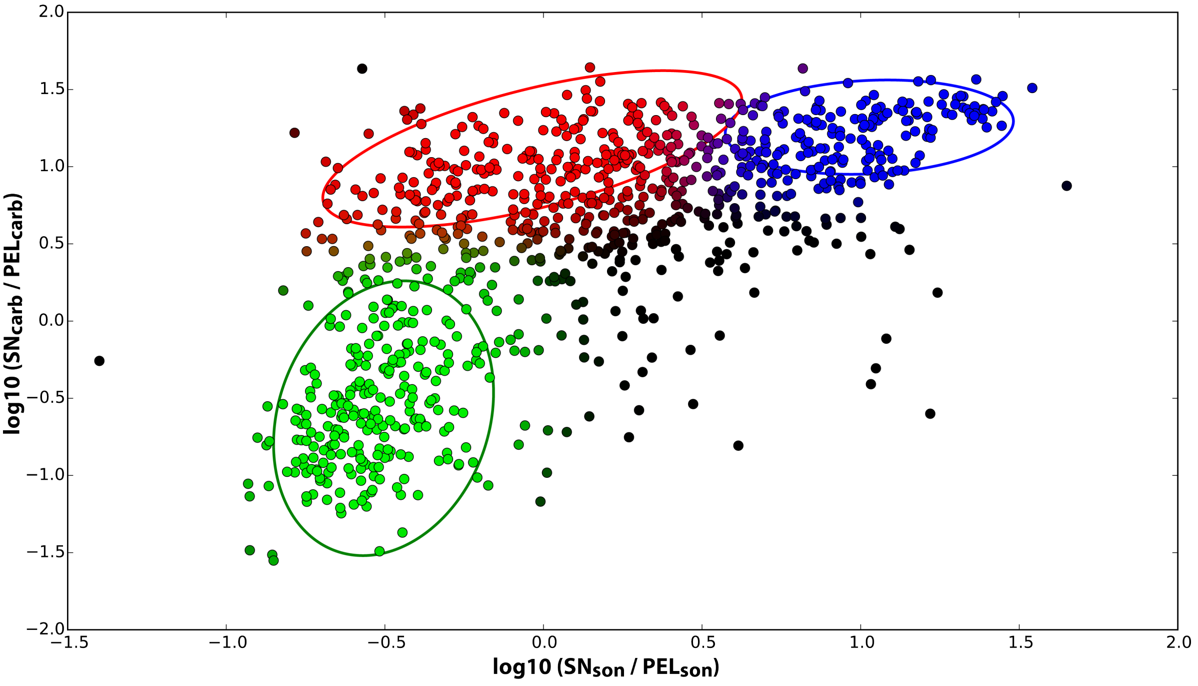

Supplementary Figure 4 | Distribution of all proteins P_L+P_U. To indicate memberships, all 3 color channels RGB were multiplied by their corresponding probabilities for every protein. The model boundaries represent 80% of each density. Integral membrane proteins (green); peripheral membrane proteins (orange); soluble proteins (blue).

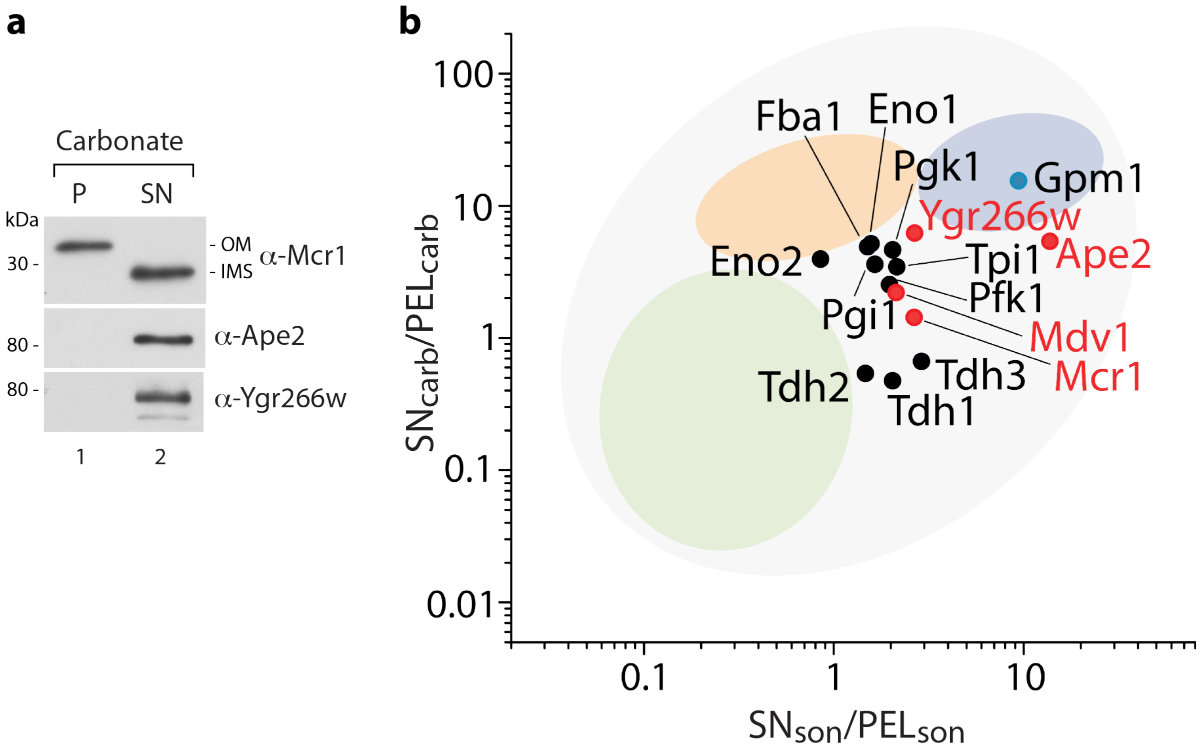

Supplementary Figure 5 | Analysis of ambiguous proteins. (**a**) Immunoblot analysis of mitochondria after carbonate extraction. P, pellet; SN, supernatant. (**b**) Distribution of selected proteins from the ambiguous map region. Black/blue, glycolysis enzymes; red, mitochondrial proteins Mdv1, Mcr1, Ape2 and Ygr266w.

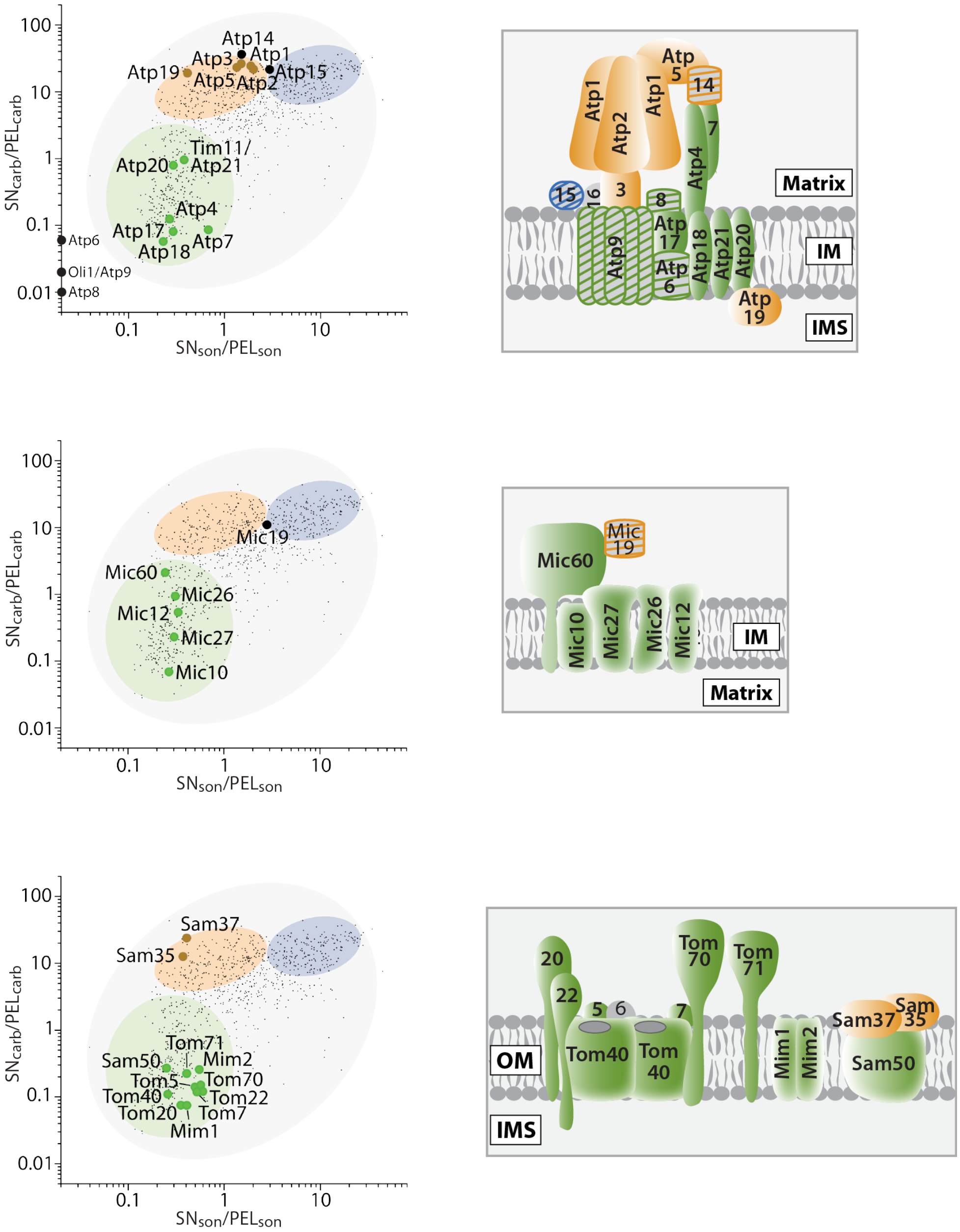

Supplementary Figure 6 | Data validation for various protein machineries. Correlation of map data with the known mitochondrial sublocalizations of components of the ATP synthase[3,4] (upper panels), the mitochondrial contact site and cristae organizing system[5] (MICOS, middle panels) and the translocase of the outer membrane (TOM complex), the mitochondrial import machinery (MIM) and the sorting and assembly machinery[6] (SAM complex, lower panels). Proteins with shaded area were manually assigned. Current models depict Atp7 as peripheral membrane protein, whereas it was identified as integral in our map. This raises the interesting possibility that Atp7 might be more tightly associated with the inner mitochondrial membrane than so far anticipated.

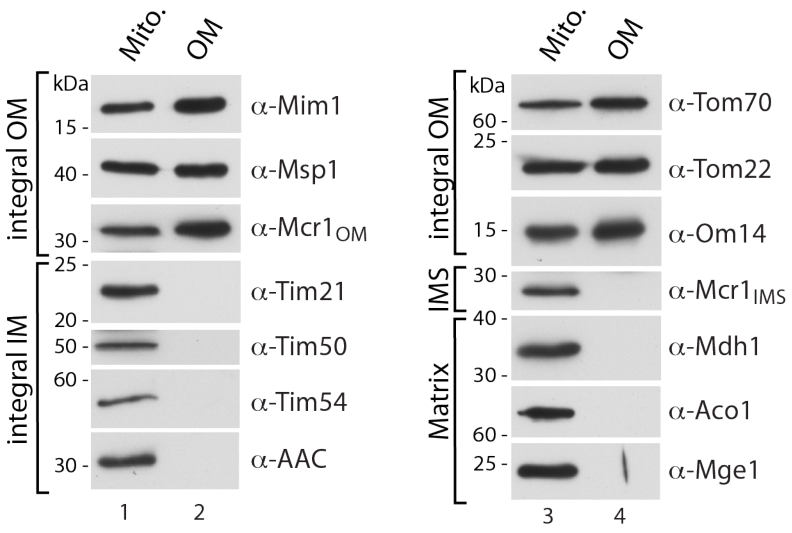

Supplementary Figure 7 l Validation of outer membrane purity by immunoblotting. Highly pure mitochondria (Mito.) and outer membrane (OM) fractions were analyzed by SDS-PAGE and immunodecoration.

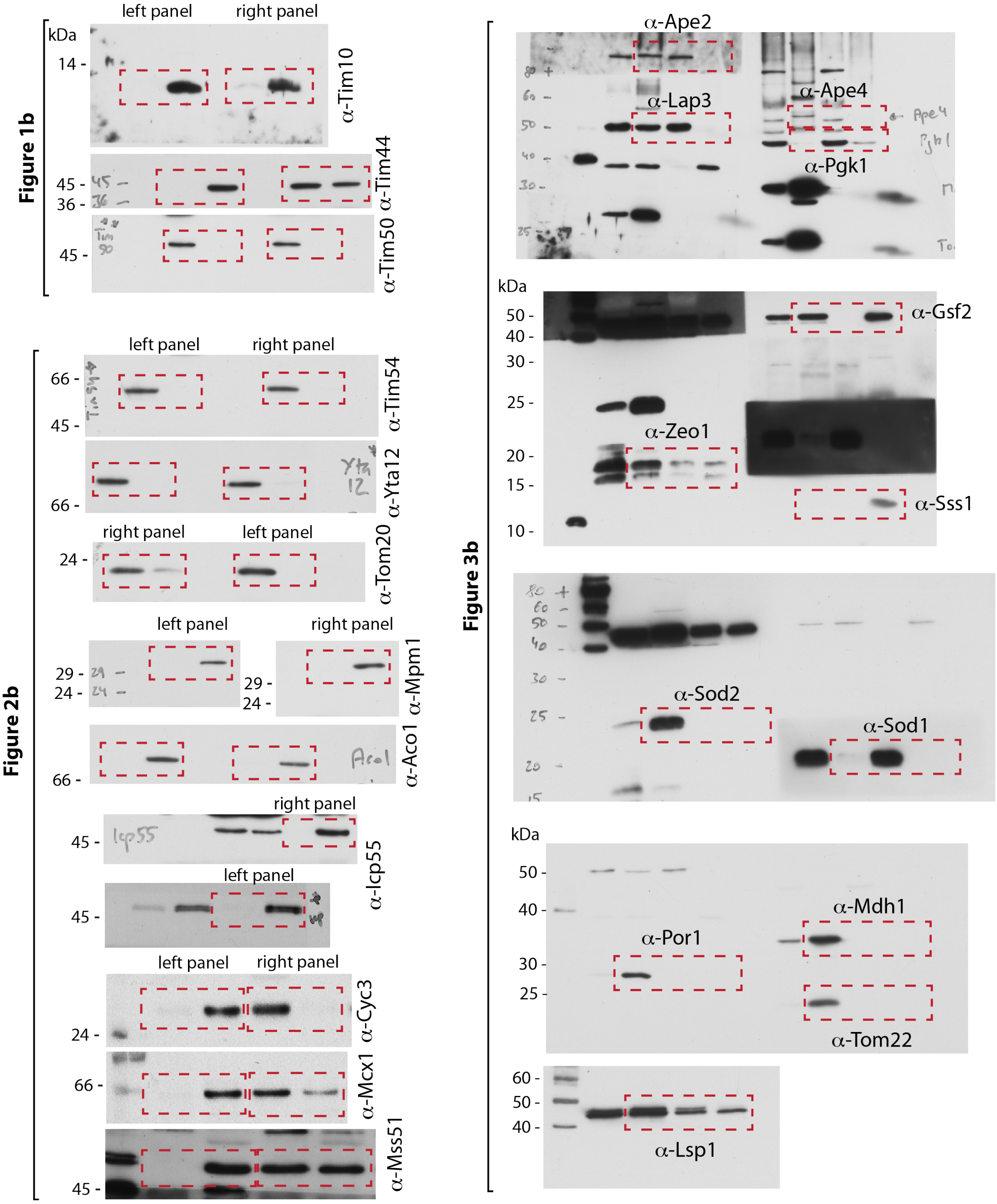

Supplementary Figure 8 | Original scans of key Western blots presented in this paper.

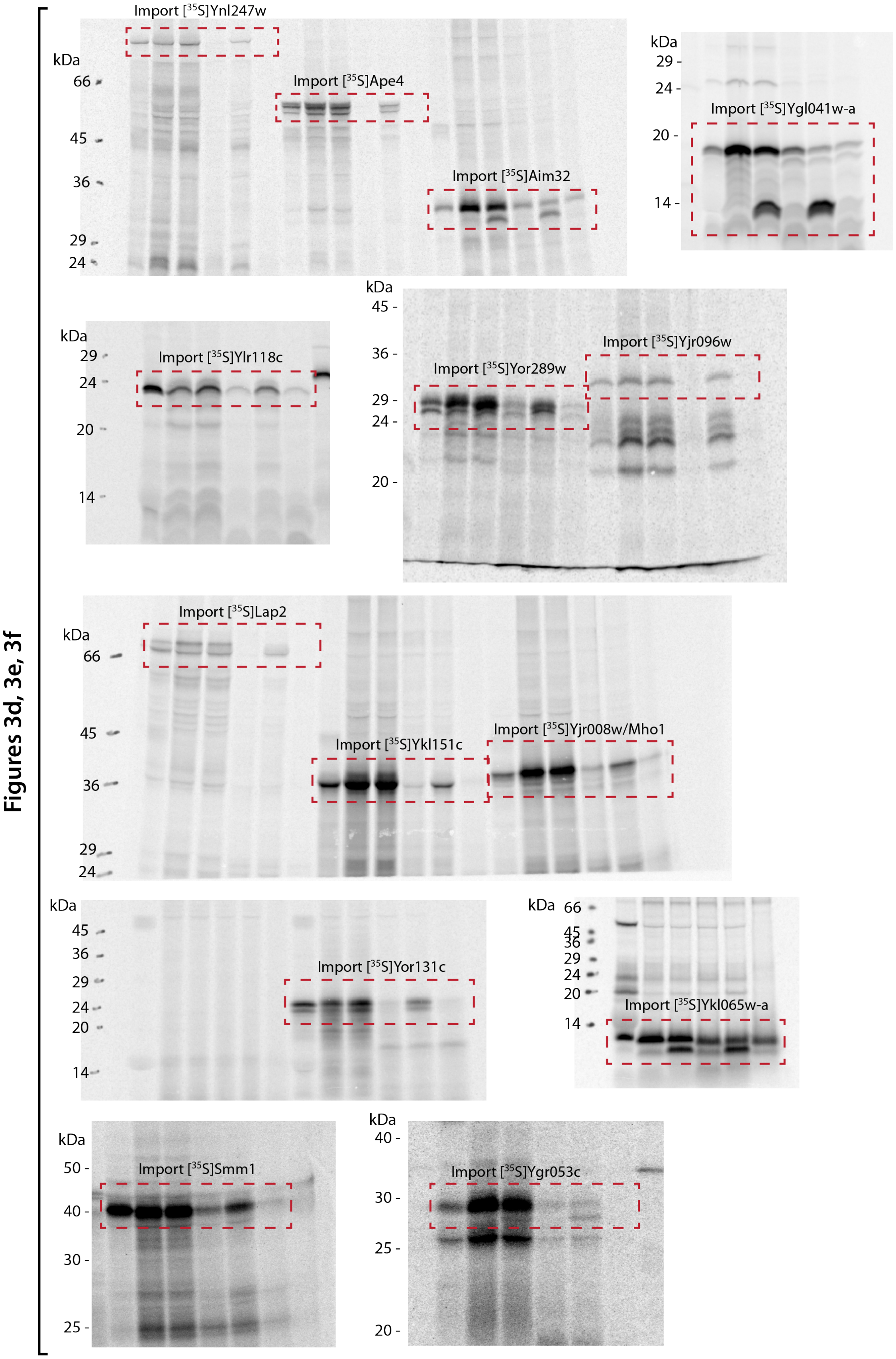

Supplementary Figure 9 | Original scans of key autoradiography gels presented in this paper.

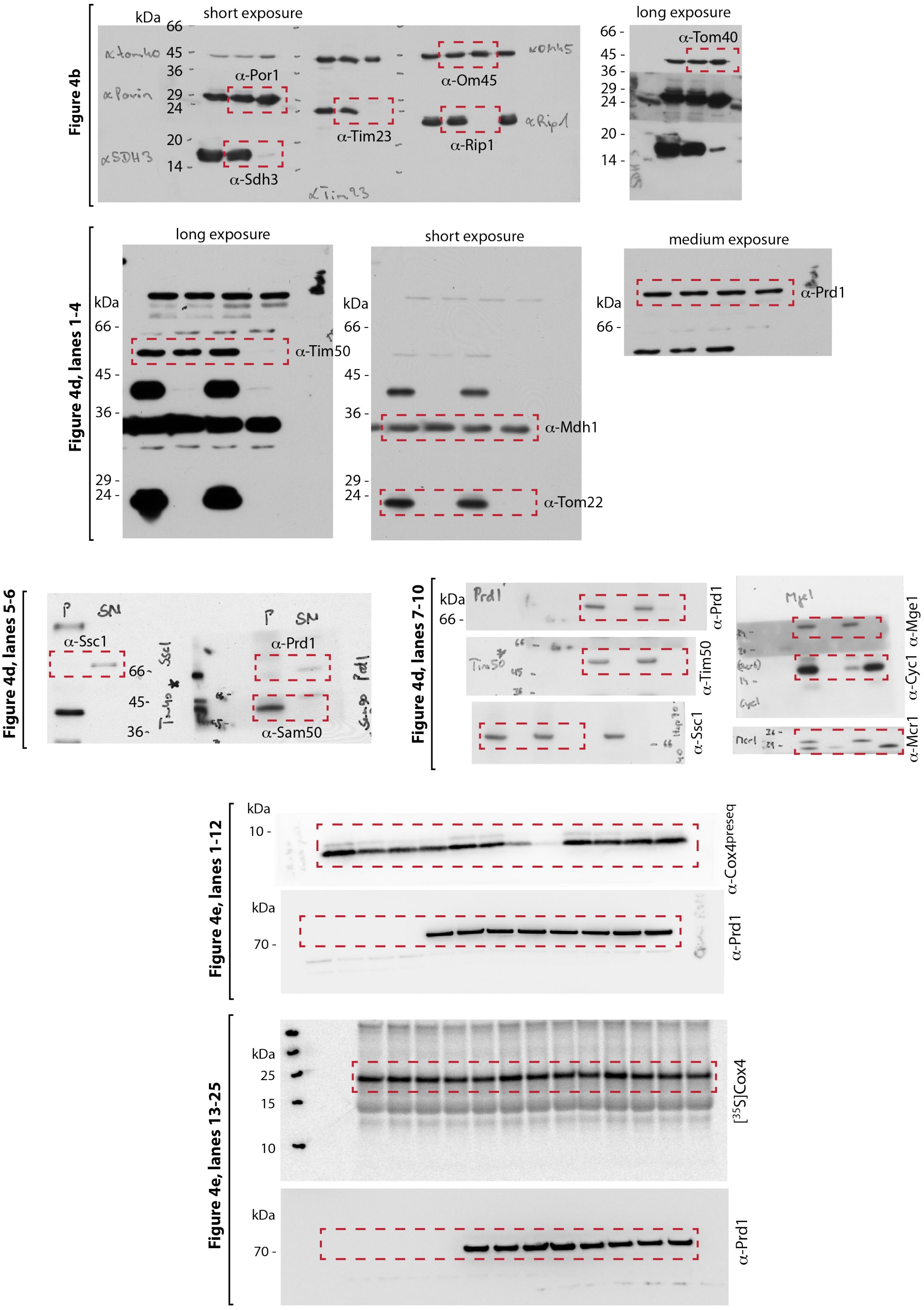

Supplementary Figure 10 | Original scans of key Western blots and autoradiography gels presented in this paper.

Supplementary Table 1: Antibodies used in this study.
All antibodies were raised in rabbits. Antisera were diluted in TBS with 5% w/v milk powder. a.p., affinity purified antiserum, diluted in TBS with 0.1% Tween20.

**Antigen**	**Dilution**	**Reference number**
Tim10	1:250	2041-7
Tim44	1:500	1835-2
Tim50	1:500	3881-1
Tim54	1:200	215-6
Yta12	1:250	1438-4
Tom20	1:5000	3225-7
Mpm1	1:500	3096-2
Aco1	1:2000	945-1
Icp55 (a.p.)	1:100	1576-4
Cyc3	1:500	620-6
Mcx1	1:1000	709-5
Mss51	1:250	1953-4
Por1	1:250	3622-2
Pgk1	1:5000	754-7
Sss1	1:1000	788-1
Sod2	1:10 000	1011-5
Mdh1	1:1000	1088-4
Tom22	1:10 000	3227-2
Lap3	1:1000	707-5
Gsf2	1:1000	1406-4
Ape4	1:250	4851-3
Ape2	1:500	4830-6
Lsp1	1:500	1376-2
Zeo1	1:1000	1481-7
Sod1	1:250	1080-5
Om45	1:2000	1390-4
Tom40	1:500	169-7
Rip1	1:500	543-6

Tim23	1:750	3878-4
Sdh3	1:1000	2438-2
Prd1	1:250	1997-7
Ssc1	1:250	41756-V2
Sam50	1:500	312-15
Cyc1	1:1000	546-2
Mcr1	1:1000	613-7
Mge1	1:1000	23210-V
Cox4$^{preseq.}$ (a.p.)	1:100	3411-4
Tom70	1:500	657-3
Tim21	1:250	3883-4
Cox6	1:2000	2015-2
Mim1	1:500	545-6
Msp1	1:1000	1468-4
AAC	1:1000	227-2
Om14	1:1000	3040-1
Atp2	1:1000	863-4
Sec61	1:500	760-7
Hsp30	1:500	720-5
Vam3	1:500	766-6
Ssa1	1:250	1011-4
Hsc82	1:500	1050-5
Bcy1	1:500	1491-5
Nma111	1:500	341-2
Ygr266w	1:500	1370-7
Nup85	1:400	gift from E. Hurt
Pex13	1:5000	gift from W. Kunau

Supplementary references

1. Chacinska, A. *et al.* Mitochondrial Presequence Translocase: Switching between TOM tethering and motor recruitment involves Tim21 and Tim17. *Cell* **129**, 817-829 (2005).
2. Song, J., Tamura, Y., Yoshihisa, T. & Endo, T. A novel import route for an N-terminal mitochondrial outer membrane protein aided by the TIM23 complex. *EMBO Rep.* **15**, 670-677 (2014).
3. Lytovchenko, O. et al. The INA complex facilitates assembly of the peripheral stalk of the mitochondrial F1F0-ATP synthase. *EMBO J.* **33**, 1624-1638 (2014).
4. Devenish, R.J., Prescott, M., Roucou, X. & Nagley, P. Insights into ATP synthase assembly and function through the molecular genetic manipulation of subunits of the yeast mitochondrial enzyme complex. *Biochim. Biophys. Acta* **1458**, 428-442 (2000).
5. Pfanner, N. *et al.* Uniform nomenclature for the mitochondrial contact site and cristae organizing system. *J. Cell Biol.* **204**, 1083-1086 (2014).
6. Schmidt, O., Pfanner, N. & Meisinger, C. Mitochondrial protein import: from proteomics to functional mechanisms. *Nature Rev. Mol. Cell. Biol.* **11**, 655-667 (2010).
