# Supplementary figures and images for: Landscape of submitochondrial protein distribution
Source: Nat Commun. 2017 Aug 18;8:290. doi: 10.1038/s41467-017-00359-0 (PMC5561175; doi:10.1038/s41467-017-00359-0)

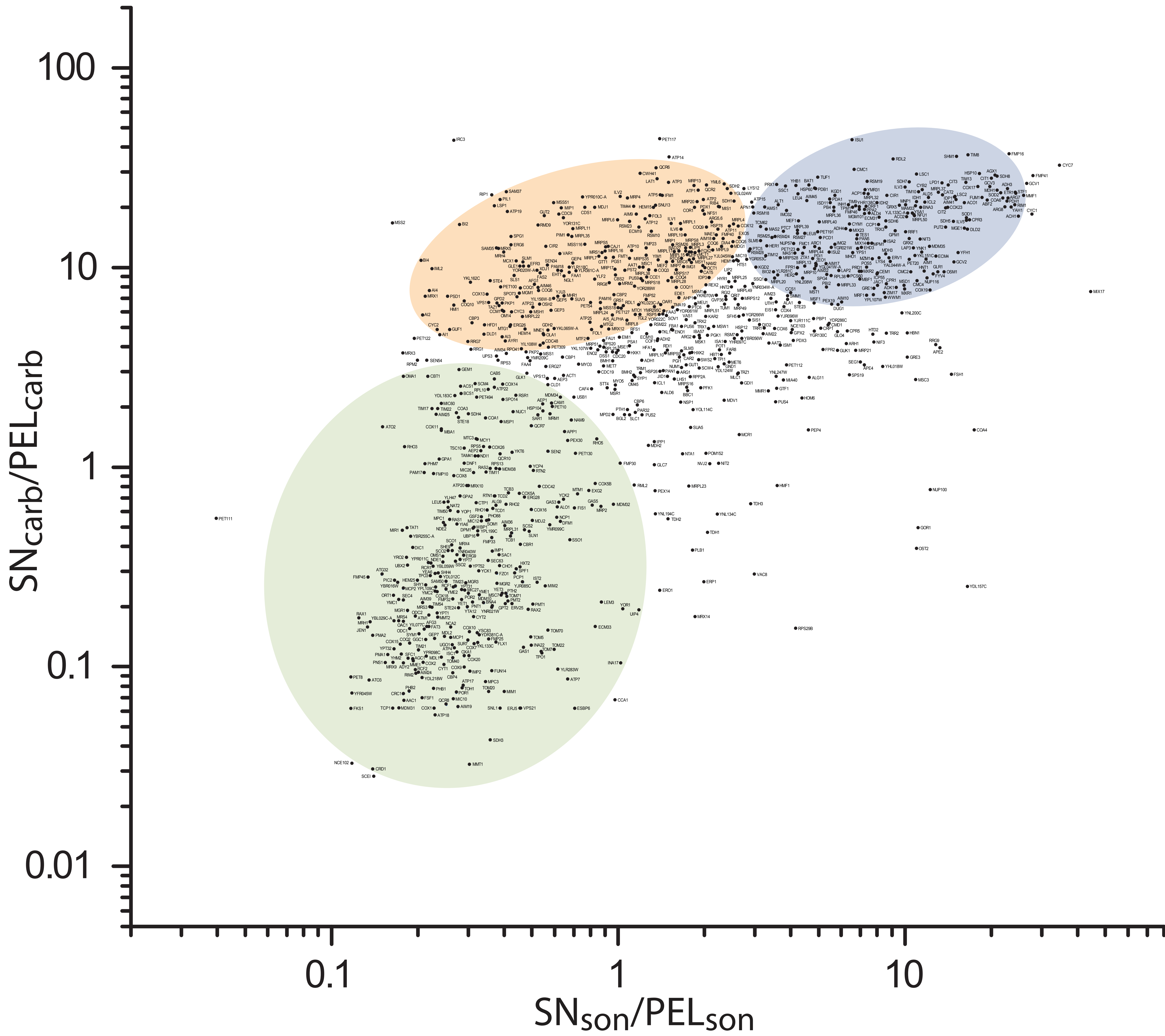

Supplement: Supplementary file 3 — Supplementary Data 1 [file 41467_2017_359_MOESM3_ESM.pdf]
